# The impact of COVID-19 infection on musculoskeletal pain and its associating factors: a cross-sectional study

**DOI:** 10.3389/fpubh.2024.1422659

**Published:** 2024-08-27

**Authors:** Hongyan Li, Shiyang Zhuang, Yiming Lin, Mei Huang, Wenming Zhang, Xuehui Zhang, Yunzhi Lin, Chaofan Zhang

**Affiliations:** ^1^Department of Orthopaedic Surgery, the First Affiliated Hospital, Fujian Medical University, Fuzhou, China; ^2^Department of Orthopaedic Surgery, National Regional Medical Center, Binhai Campus of the First Affiliated Hospital, Fujian Medical University, Fuzhou, China; ^3^Fujian Provincial Institute of Orthopedics, the First Affiliated Hospital, Fujian Medical University, Fuzhou, China; ^4^Fujian Orthopedic Bone and Joint Disease and Sports Rehabilitation Clinical Medical Research Center, Fuzhou, China; ^5^Department of Stomatology, the First Affiliated Hospital, Fujian Medical University, Fuzhou, China; ^6^Department of Stomatology, National Regional Medical Center, Binhai Campus of the First Affiliated Hospital, Fujian Medical University, Fuzhou, China; ^7^School of Stomatology, Fujian Medical University, Fuzhou, China; ^8^School of Public Health, Fujian Medical University, Fuzhou, China; ^9^School of Health Management, Fujian Medical University, Fuzhou, China

**Keywords:** musculoskeletal pain associated with COVID-19 (MSPC), health-seeking behavior, cross-sectional study, pandemic, COVID-19

## Abstract

**Objectives:**

Musculoskeletal pain after COVID-19 infection remains a concerning long-term complication of COVID-19. Here, our study aimed to investigate the prevalence of musculoskeletal pain associated with COVID-19 (MSPC) and healthcare-seeking behaviors, as well as the associating factors.

**Methods:**

A cross-sectional survey was conducted using convenience sampling and distributed to participants anonymously through the online platform Credamo. Demographic and characteristic data of the participants were collected and analyzed. Logistic regression analysis was employed to investigate potential factors associated with MSPC and healthcare-seeking tendencies.

**Results:**

A total of 1,510 participants responded to the survey, with 42.6% (643 individuals) exhibiting MSPC. Higher education level and a greater number of concomitant symptoms were significant risk factors for MSPC, while longer exercise duration and higher PSS-10 scores were protective factors. Additionally, higher income level, frequency and severity of pain, and greater PSS-10 scores increased healthcare-seeking intention.

**Conclusion:**

A significant proportion of individuals experience MSPC. Education level and concomitant symptoms were risk factors for MSPC, while exercise duration and PSS-10 score were potential protective factors. Income level, frequency and severity of pain, and PSS-10 score are significantly related to the willingness to seek medical treatment for MSPC.

## Introduction

Since the outbreak of the COVID-19 epidemic in Wuhan, China in 2019, the coronavirus has become a global threat to citizens. The Omicron variant first appeared in November 2021 in South Africa, which has emerged as the predominant strain worldwide, displaying a higher transmission rate and milder symptoms of infection (1). According to data from the Chinese Center for Disease Control and Prevention, from September 26, 2022, to February 23, 2023, a total of 27,315 locally transmitted cases of COVID-19 with valid genomic sequences of the Omicron variant were reported nationwide, encompassing 80 evolutionary branches (2). Despite the apparent reduction in the virus’s harmful effects, a multitude of persistent symptoms, including cough, fever, difficulty breathing, gastrointestinal symptoms, as well as olfactory dysfunction and musculoskeletal symptoms have emerged. Multiple organ systems could be involved in viral infection in addition to respiratory symptoms and pulmonary manifestations (3). Given the unclear mechanisms related to viral pathogenicity, damage to endothelial cells, thrombotic events, and disruptions in the Renin-Angiotensin-Aldosterone System (RAAS) (4), there is an urgent need for in-depth investigation to better comprehend these processes.

Musculoskeletal pain is a prevalent condition experienced by many individuals throughout their lives, potentially affecting around 47% of the global population. It is also a common reason for seeking primary healthcare. The costs of treating musculoskeletal pain and the resulting loss of productivity can significantly impact patients’ quality of life and become a substantial socioeconomic burden (5). During COVID-19 infection, musculoskeletal pain is a common symptom, involving pain in bones, joints, muscles, tendons, and ligaments (5). Recent research suggests that after the initial COVID-19 infection, musculoskeletal pain will continue to exist (6). The incidence of this symptom varies globally, with approximately 6.2 to 30% (7, 8). However, there is limited study that has focused only on the development of musculoskeletal pain associated with COVID-19 (MSPC) and its associated risk factors in the general population.

Actually, the manifestation of musculoskeletal symptoms varied among different strains of the virus. Following infection with the China historical strain, approximately 50% of patients experienced *de novo* musculoskeletal pain symptoms post-COVID (9). The literature indicates that COVID-19 can trigger musculoskeletal pain in the population and alter pre-existing pain symptoms in patients, with different strains having varying impacts on musculoskeletal pain (9). However, during the pandemic, individuals might avoid visiting their general practitioner because they might be concerned about the risk of contracting COVID-19 (10). Although many symptoms in primary care are self-limiting, it is crucial to seek timely medical evaluation for some conditions to prevent health deterioration. At present, there is limited literature research on the healthcare-seeking behavior of musculoskeletal pain specifically associated with the omicron variant as the predominant strain.

Therefore, this study aims to investigate the prevalence of MSPC during the omicron outbreak through a cross-sectional study and explore its potential associating factors. Additionally, we will further analyze factors that are related to healthcare-seeking behavior among individuals with MSPC.

## Methods

### Study design

The study employed a cross-sectional design with a self-administered survey to assess the prevalence and potential associating factors of musculoskeletal pain associated with COVID-19 (MSPC) and health-seeking behavior. This approach allowed participants to complete the questionnaire independently ensuring flexibility and accessibility for a diverse group of respondents.

### Setting

Participants were recruited and completed a validated questionnaire assessing general demographic characteristics, COVID-19 infection status, musculoskeletal symptoms, impact on daily activities, and psychological well-being.

### Variables

Musculoskeletal pain is defined as chronic pain in the muscles, bones, joints, or tendons, characterized by significant emotional distress (such as anxiety, anger, frustration, and sadness) or functional impairment. The definition of MSPC is as follows: participants report the development of new musculoskeletal pain following COVID-19 infection or an increase in the severity and frequency (exacerbation) of pre-existing pain. Muscle pain is defined as myalgia which can involve ligaments, tendons, and fascia, the soft tissues that connect muscles, bones, and organs. Neck pain is pain that starts in the neck and can be associated with radiating pain down one or both of the arms. Back pain is defined as pain, muscular tension, or stiffness that is localized at the upper, middle, or lower back. Joint pain is defined as discomfort, aches, and soreness in any of the body’s joints.

### Ethical considerations

This study has been approved by the Institutional Ethics Committee of the First Affiliated Hospital of Fujian Medical University [MRCTA, ECFAH of FMU [2023] 307].

### Participant selection criteria

**Figure 1:**
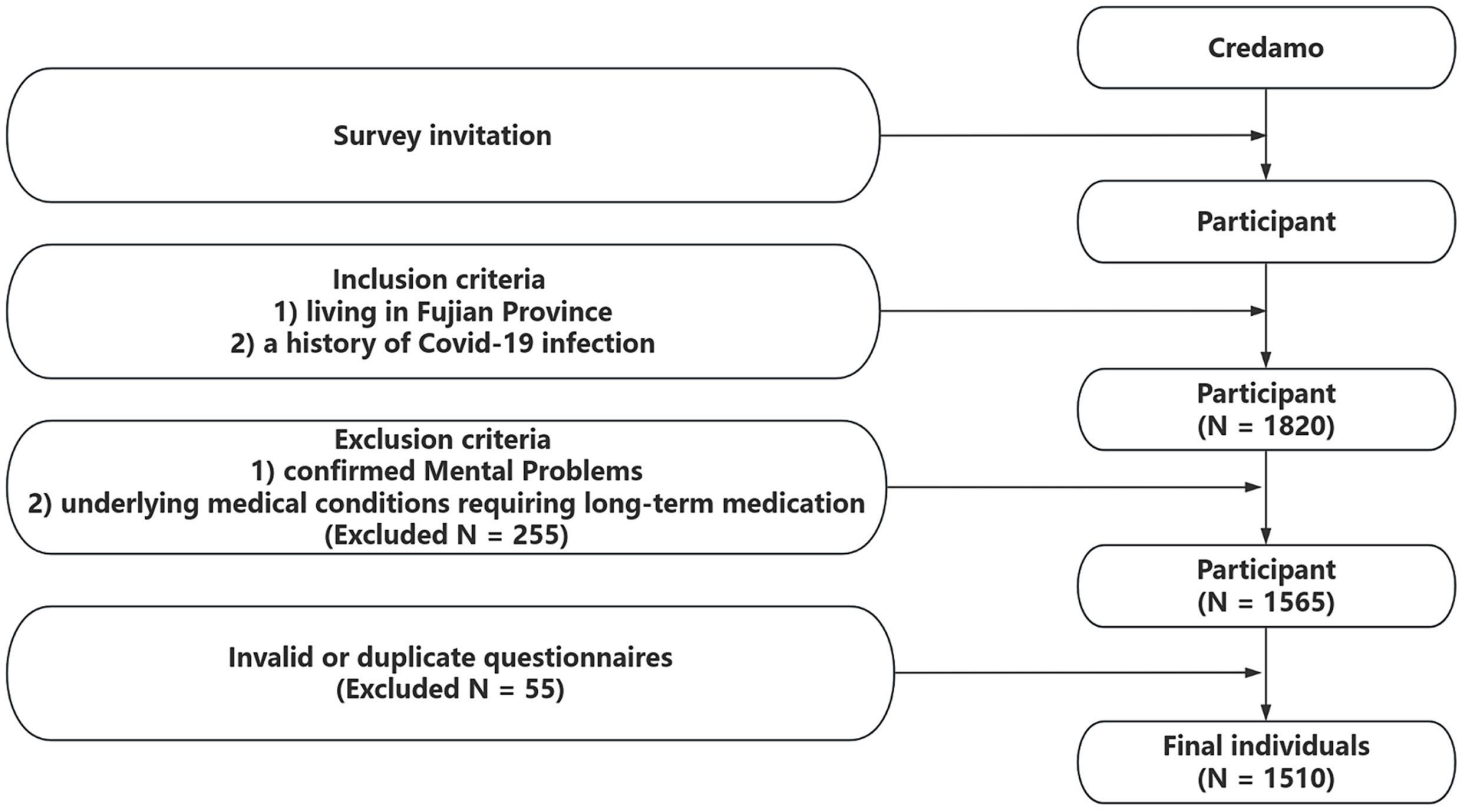
The flowchart of recruitment.

The target population for our survey study includes individuals who meet the following criteria: (1) a history of COVID-19 infection and (2) currently residing in the Fujian province at the time of questionnaire collection. The following individuals are excluded from the survey: (1) those with a documented history of mental and psychological disorders before the onset of the pandemic, and (2) individuals with underlying medical conditions requiring long-term medication, such as coronary heart disease, stroke, hypertension, chronic obstructive pulmonary disease (COPD), diabetes, chronic kidney disease, cancer, human immunodeficiency virus (HIV)/acquired immunodeficiency syndrome (AIDS). Participants with mental or psychological issues were not included in these groups due to the well-established link between such conditions and heightened pain sensitivity (11). Consistent with this approach, previous similar research has also opted to exclude individuals with a history of diagnosed mood disorders (12) (Figure 1).

### Data collection

Participants were invited to complete the questionnaire online through the Credamo platform. Credamo is a professional data platform with a sample database of more than 1.5 million participants, which can provide large-scale data collection services and has been recognized by international top journals in the fields of psychology, management, sociology, and environmental science (13). To further encourage participation, we implemented a compensation mechanism on the platform, aiming to boost the motivation of potential respondents. The participants would receive CNYҰ5 (US $0.75) for incentives.

### Sample size

The study required a sample size of 384 individuals, which was determined by distributing anonymous questionnaires to the general public through the Credamo platform. The calculation was based on a formula $n=Z^{2}\frac{P\left(1-P\right)}{d^{2}}$ , considering a 95% confidence interval, a preliminary small-scale survey indicating a 50% prevalence rate of symptoms, and a margin of error of 0.05 (11). Due to the methodology for sample collection in this study, the sample size can be multiplied by a factor of 2 to account for the anticipated design effect. Therefore, the minimum required sample size for the study is 768 individuals (384 × 2).

### Questionnaire

The questionnaire was developed to comprehensively assess various aspects related to musculoskeletal pain, encompassing demographic characteristics, COVID-19 infection status, musculoskeletal pain symptoms, impact on daily activities, and psychological status and its association with COVID-19 infection. The questionnaire included standardized questions related to MSPC and its associated features, as well as the willingness of people to seek medical care after suffering from MSPC. Informed consent was obtained from all participants prior to study participation, with strict maintenance of privacy and confidentiality.

The questionnaire consisted of six sections. The first section collected general information, including age, gender, height, weight, ethnicity, smoking/alcohol history, education level, income level, and daily habits. The second section assessed the participants’ musculoskeletal pain status before the COVID-19 infection, including pain location, pain frequency, pain intensity, and measures taken to manage the pain. The Visual Analog Scale (VAS) was used to measure the severity of pain, ranging from 0 (no pain) to 10 (most severe pain). The response options for pain frequency were “rarely, sometimes, often, very frequent, and persistent.” Participants reporting any self-reported pain symptoms were categorized as the “pain group.” The third section investigated the participants’ COVID-19 infection status, including the time and method of diagnosis, the number of vaccine doses received, the overall duration of illness, medications taken during the infection, and other symptoms experienced besides pain (concomitant symptoms, including nasal congestion, fatigue, dizziness, sore throat, reduced sense of smell, chest tightness, diarrhea, skin rashes, papules, herpes, and oral ulcers). The fourth section explored the participants’ MSPC, including pain frequency, intensity, and measures taken to manage the pain including their willingness to seek medical care. The fifth section assessed the participants’ knowledge of musculoskeletal diseases, including their understanding of disease knowledge, etiology, symptomatology, and treatment. The final section consisted of the Perceived Stress Scale-10 (PSS-10) questionnaire, which evaluated the participants’ psychological status during the COVID-19 pandemic.

The questionnaire content was previously evaluated by a panel of experts from the fields of pain medicine, psychology, and health sciences. Following this evaluation, we conducted a pilot test with 30 individuals outside the final study sample to gauge the difficulty of completion, after which the questionnaire underwent assessments for both reliability and validity. The first section collected basic information about the participants. The second section had a reliability coefficient (Cronbach’s *α*) of 0.881 and a validity coefficient (Kaiser–Meyer–Olkin test, KMO coefficient) of 0.815. The third section assessed the participants’ infection status. The fourth section had a reliability coefficient of 0.908 and a validity coefficient of 0.875. The fifth section had a reliability coefficient of 0.856 and a validity coefficient of 0.825. The sixth section had a reliability coefficient of 0.613 and a validity coefficient of 0.794. These data indicate that the questionnaire exhibited good reliability and validity. After completing the survey, the data were organized and entered into a password-protected database.

### Statistical analysis

The data collected through our survey were analyzed to determine the relationships and associations on MSPC during the COVID-19 pandemic. Initially, we assessed the distribution of numerical variables to ascertain the applicability of parametric statistical tests. Normality was confirmed using Q–Q plots and the Shapiro–Wilk test, ensuring the data met the assumptions for the subsequent analyses.

Quantitative data were presented as “mean ± standard deviation,” and qualitative data were presented as frequencies. The chi-square test was used for analyzing qualitative data, while the *t*-test was used for normally distributed continuous data. To measure the strength and direction of the linear relationship between continuous variables, we calculated Pearson’s Product Moment Correlation Coefficient, providing insight into potential correlations.

Further, to identify factors related to MSPC during the COVID-19 period and participants’ healthcare-seeking behavior, we performed univariate and multivariate logistic regression analyses. Variables significant in the univariate analysis (two-tailed, *p*-value <0.1) were selected for inclusion in the multivariate model using a forced entry method. For each independent variable, we calculated Odds ratios (OR), 95% confidence intervals (CI), and *p*-values, with statistical significance defined as *p* < 0.05. Additionally, the Hosmer–Lemeshow goodness-of-fit test was used to evaluate the model fit. If the significance value was <0.05, the Hosmer–Lemeshow statistic indicated a poor fit. All of these analyses were conducted using SPSS version 28.0 (IBM Corp.).

## Results

### Demographics

The questionnaire was distributed to 1,820 subjects, and a total of 1,565 subjects responded to the survey, with a response rate of 85.9%. Among the questionnaires received, based on the inclusion/exclusion criteria, 1,510 questionnaires were finally included in this study. The participants had an average age of 32.04 ± 10.13 years, with 615 males and 895 females. The average BMI was 22.79 ± 5.72. The majority of participants, 1,484 individuals (98.3%), were of Han ethnicity. Additionally, a large proportion of participants (over 80%) had attained a bachelor’s degree or higher. Furthermore, 18.6% of participants were smokers, and 24.4% were drinkers. Table 1 provides an overview of the participant’s demographic characteristics.

**Table 1:** The demographic characteristics of participants.

Variables	Total	MSPC	Non-MSPC (%)	*χ*^2^ or *F*	*P*-value
Number of participants	1,510	643 (42.6)	867 (57.4)		
Age	32.04 ± 10.03	33.59 ± 10.62	29.95 ± 9.03		<0.001
Gender				0.93	0.33
Male	615 (40.7)	271 (42.1)	344 (39.7)		
Female	895 (59.3)	372 (57.9)	523 (60.3)		
Ethnicity				1.37	0.24
Han	1,484 (98.3)	629 (97.8)	855 (98.6)		
Others	26 (1.7)	14 (2.2)	12 (1.4)		
BMI	22.79 ± 5.72	23.07 ± 5.83	22.58 ± 5.63	0.93	0.09
Educational level				25.73	<0.001
Junior High School or Below	41 (2.7)	5 (0.8)	36 (4.2)		
High School	214 (14.2)	72 (11.2)	142 (16.4)		
University and above	1,255 (83.1)	566 (88.0)	689 (79.5)		
Incomes level (CNY)				17.08	<0.001
<2000Ұ/m	267 (17.7)	136 (21.2)	131 (15.1)		
2000–7,000Ұ/m	412 (27.3)	191 (29.7)	221 (25.5)		
>7,000Ұ/m	831 (55.0)	316 (49.1)	515 (59.4)		
Drinking				14.28	0.003
Non-drinker (0 g/d)	1,142 (75.6)	503 (78.2)	639 (73.7)		
Mild drinker (0.1–9.9 g/d)	241 (16.0)	106 (16.5)	135 (15.6)		
Moderate drinker (10.0–29.9 g/d)	122 (8.1)	33 (5.1)	89 (10.3)		
Heavy drinker (>50.0 g/d)	5 (0.3)	1 (0.2)	4 (0.5)		
Smoking				51.84	<0.001
0 cigarette/d (non-smoker)	1,229 (81.4)	543 (84.4)	686 (79.1)		
0–5 cigarettes/d	141 (9.3)	77 (12.0)	64 (7.4)		
5–15 cigarettes/d	108 (7.2)	17 (2.6)	91 (10.5)		
15–20 cigarettes/d	26 (1.7)	3 (0.5)	23 (2.7)		
>20 cigarettes/d	6 (0.4)	3 (0.5)	3 (0.3)		

MSPC, musculoskeletal pain associated with COVID-19; CNY, Chinese Yuan; BMI, Body Mass Index.

### The characteristics of individuals with MSPC

Among the participants, a total of 643 (42.6%) individuals exhibited MSPC. As shown in Table 1, there are several notable differences between the group experiencing MSPC and the group without MSPC: the former group had a significantly older age, higher educational level, lower income level, a higher proportion of smokers, and a higher proportion of drinkers. There were no significant differences between the two groups in terms of gender, ethnicity, and BMI.

### Prevalence of MSPC

**Figure 2:**
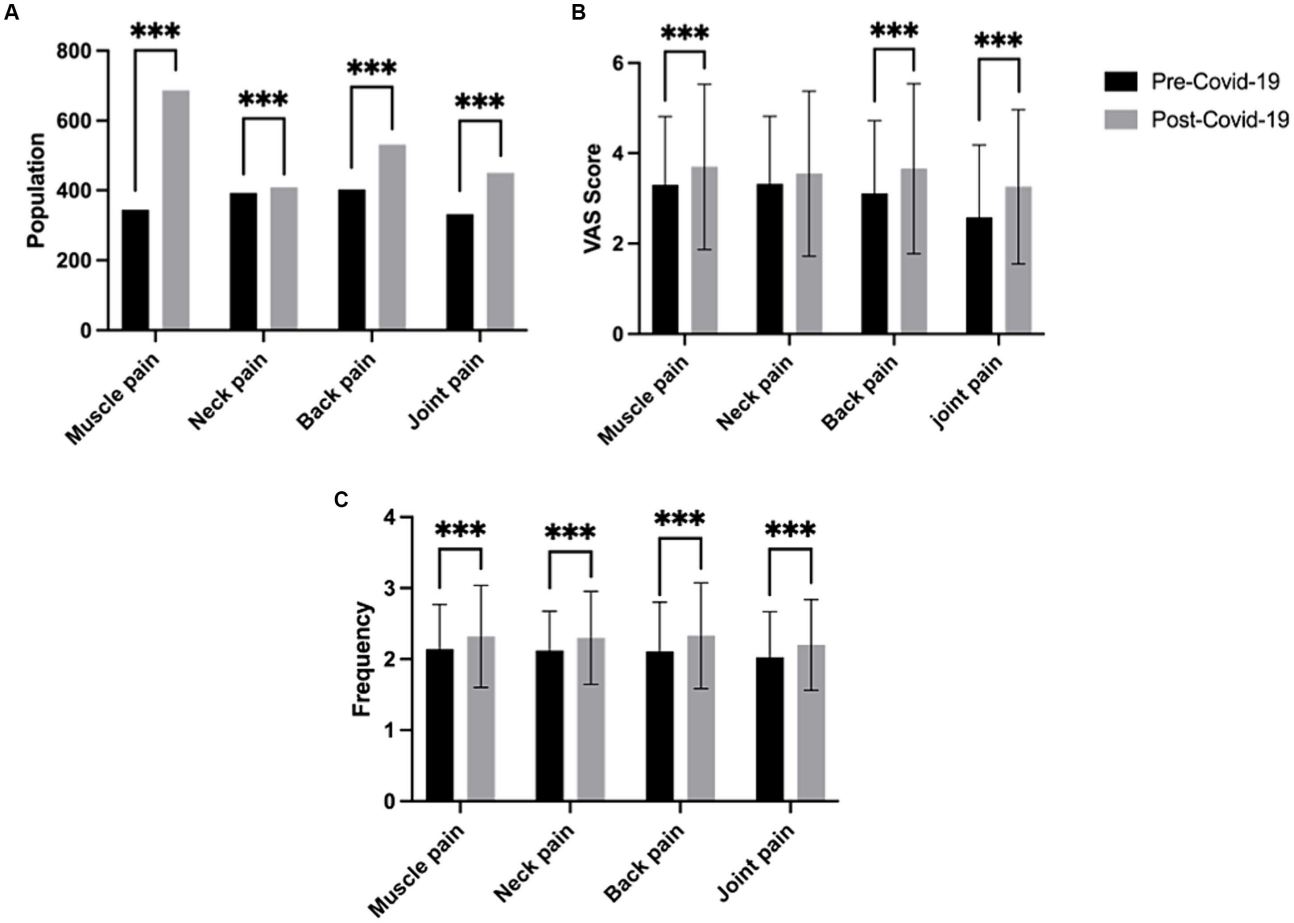
Comparison of the number, severity, and frequency of musculoskeletal pain before and after COVID-19. **(A)** Number of individuals with different types of musculoskeletal pain. **(B)** Comparison of the severity of different types of musculoskeletal pain before and after COVID-19. **(C)** Comparison of the frequency of different types of musculoskeletal pain before and after COVID-19. (*** indicates statistical significance with *p* < 0.001).

A total of 806 (53.37%) participants reported experiencing musculoskeletal pain before COVID-19 infection. After the COVID-19 infection, the number of participants reporting musculoskeletal pain increased to 991 (65.22%). The comparison of different types of pain before and after COVID-19 infection is shown in Figure 2. The number of individuals experiencing various types of musculoskeletal pain significantly increased (Figure 2A). Specifically, the number of individuals with muscle pain increased from 345 (23.05%) to 687 (46.03%). Neck pain increased from 393 (26.36%) to 409 (27.42%). While back pain increased from 403 (27.02%) to 532 (35.70%). Joint pain also increased from 332 (22.38%) to 450 (30.26%). Furthermore, the VAS scores for musculoskeletal pain also increased after COVID-19 (Figure 2B). The VAS score for muscle pain increased from 3.30 ± 1.52 to 3.70 ± 1.83, neck pain increased from 3.32 ± 1.50 to 3.55 ± 1.83, back pain increased from 3.11 ± 1.62 to 3.66 ± 1.88, and joint pain increased from 2.58 ± 1.61 to 3.26 ± 1.71. The frequency of pain in different body regions also increased (Figure 2C). Muscle pain was significantly increased from 2.14 ± 0.63 to 2.32 ± 0.72, neck pain from 2.12 ± 0.5 to 2.30 ± 0.65, back pain from 2.11 ± 0.69 to 2.33 ± 0.74, joint pain from 2.03 ± 0.64 to 2.20 ± 0.64.

### The associated factors of the MSPC

As shown in Table 2, the univariable logistic regression analysis revealed that age, smoking, alcohol consumption, education level, exercise, sleep, standing, sedentary behavior, walking, duration of electronic device use, PSS-10 score, and the number of concomitant symptoms may be associated with MSPC.

**Table 2:** The factors associated with the MSPC.

Variables	Univariable analysis (*P*-value)	Multivariable analysis (*P*-value)	OR [95% CI]
Gender	0.334		
Age	<0.001	0.841	1.015 [0.876–1.177]
BMI	0.1		
Ethnicity	0.245		
Smoking	<0.001	0.5	0.929 [0.752–1.149]
Drinking	0.002	0.746	0.964 [0.773–1.202]
Educational level	<0.001	<0.001	2.004 [1.443–2.784]
Income level	<0.001	0.784	0.973 [0.803–1.181]
Lifestyle			
Exercise	<0.001	<0.001	0.569 [0.486–0.667]
Sleep	0.015	0.192	0.917 [0.804–1.045]
Standing	0.001	0.73	1.020 [0.913–1.139]
Sit (study or work)	<0.001	0.995	1.000 [0.933–1.072]
Walk	<0.001	0.914	1.008 [0.874–1.163]
Electronic devices usage	<0.001	0.667	0.978 [0.882–1.083]
PSS-10	0.082	0.007	0.962 [0.936–0.990]
Vaccination status	0.837		
Duration of COVID-19	0.99		
Number of concomitant symptoms	<0.001	<0.001	1.538 [1.418–1.667]

OR, odds ratios; 95%CI, 95% confidence intervals; BMI, Body Mass Index; PSS-10, The Perceived Stress Scale.

The multivariable logistic regression analysis indicated that education level, exercise duration, PSS-10 score, and the number of concomitant symptoms were significant factors associated with MSPC. Specifically, a higher education level and a higher number of concomitant symptoms may be risk factors, while longer exercise duration and higher PSS-10 scores were identified as protective factors.

### The associated factors affecting the intention of seeking medical treatment

In this study, it was found that only 77 individuals (0.08%) among the population experiencing MSPC sought medical attention. To further investigate the factors associated with participants’ healthcare-seeking behavior, we conducted univariable and multivariable logistic regression analyses on several risk factors. The univariable logistic regression analysis showed that gender, education level, income level, PSS-10 score, pain severity, pain frequency, and disease awareness were potential factors for participants’ healthcare-seeking behavior (Table 3).

**Table 3:** The factors associated with the intention of seeking medical treatment.

Variables	Univariable analysis (*P*-value)	Multivariable analysis (*p*-value)	OR [95% CI]
Age	0.719		
Gender	0.028	0.118	0.608 [0.326–1.134]
Educational level	0.027	0.853	0.914 [0.354–2.359]
Income level	<0.001	<0.001	3.929 [2.255–6.845]
Degree of pain	<0.001	<0.001	1.215 [1.130–1.306]
Frequency of pain	<0.001	<0.001	1.337 [1.175–1.521]
Degree × Frequency	<0.001	<0.001	0.992 [0.988–0.996]
Disease awareness	0.065	0.106	1.112 [0.978–1.264]
PSS-10	<0.001	0.001	1.122 [1.050–1.200]

OR, odds ratios; 95%CI, 95% confidence intervals; PSS-10, The Perceived Stress Scale.

### The correlation between different variables

The relationship between different variables is an ambiguous issue. Thus, we conduct the strength and direction of a relationship between gender and drinking, age and electronic device usage, PSS-10, and healthcare-seeking intention by associative analysis. The result revealed that gender and drinking showed a strong positive correlation, while age and electronic device usage showed a negative correlation. PSS-10 and healthcare-seeking intention also showed a positive correlation (Supplementary Table S1).

### The factors associated with the *ex novo* musculoskeletal pain and the exacerbated musculoskeletal pain after COVID-19 infection-Second order level

In our study, we found that 643 individuals (42.6%) experienced musculoskeletal pain during the Omicron epidemic, while 374 individuals (24.8%) had *ex novo* musculoskeletal pain after COVID-19 infection. Additionally, 269 individuals reported an aggravation of original musculoskeletal pain after COVID-19 infection. The MSPC in our study includes the *ex novo* musculoskeletal pain after COVID-19 infection and the exacerbation of musculoskeletal pain after COVID-19. Thus, to explore the different influencing factors among them, we conducted further analysis of the study population (Supplementary Tables S2, S3).

## Discussion

This is a cross-sectional survey among residents of Fujian Province, China, using an online platform to investigate the prevalence of MSPC and its associating factors. We found that a significant proportion of individuals experience MSPC. Education level and concomitant symptoms were potential risk factors for MSPC, while exercise duration and PSS-10 score were potential protective factors. Income level, frequency and severity of pain, and PSS-10 score are significantly related to the willingness to seek medical treatment for MSPC.

After COVID-19 infection, individuals may experience a wide range of persistent health issues, which can last for weeks, months, or even years (4). Anyone infected with the COVID-19 virus can develop post-COVID symptoms, including musculoskeletal pain (14). As one of the primary post-infection symptoms, our study revealed a significant increase in the number of individuals experiencing MSPC, which is consistent with findings from multiple studies. A retrospective multi-center study conducted in Spain showed that 25.1% of COVID-19 survivors experienced worsening pain (15). Other literature has also indicated that major symptoms among Chinese COVID-19 survivors after discharge include muscle weakness (63%) and anxiety (23%) (16). Delving further into the potential mechanisms behind skeletal muscle pain following COVID-19, it may involve a variety of biological and psychological factors (15). In this situation where the mechanisms are unclear, identifying high-risk individuals is very meaningful and helps guide prevention and treatment.

Our investigation revealed an unexpected role of education level in MSPC. Results indicate that higher education levels are more likely to experience MSPC (*p* < 0.001). This may be attributed to improper work posture and prolonged posture among white-collar workers with higher education levels, as well as their higher work stress and limited rest time (*p* < 0.05) (17). Therefore, individuals with higher education levels should pay more attention to physical activity and adequate rest. Interestingly, we also found that people with higher education levels are more likely to suffer from *ex novo* musculoskeletal pain after COVID-19 infection (Supplementary Table S2). This insight serves as a call to action for those in the higher academia to seek balance through movement and rest.

Furthermore, the number of concomitant symptoms during the COVID-19 period is also associated with the manifestation of MSPC. COVID-19 concomitant symptoms include nasal congestion, fatigue, dizziness, and sore throat, among others. Our research suggests that a higher number of COVID-19 symptoms during the infection period is associated with an increased likelihood of MSPC (*p* < 0.001). However, the specific mechanisms of the association are still unclear, and changes in neurotransmitter levels, the release of inflammatory cytokines, and even psychological factors may all have an impact on this association (18).

During the COVID-19 pandemic, the overall mental health quality of the population declined significantly, accompanied by increased stress levels (19). Individuals with higher stress levels tend to experience more anxiety, which may contribute to a heightened perception of pain (20). Our research suggests that they are more willing to seek treatment, indicating that this subgroup may be less likely to experience MSPC due to their proactive pursuit of further treatment measures. We also found stress plays an important role in the exacerbation of musculoskeletal pain. Higher PSS-10 scores are associated with a lower likelihood of exacerbating musculoskeletal pain after COVID-19 (Supplementary Table S3). Thus, we conclude that stress may have a protective effect on the prevalence of MSPC. The detailed mechanism of stress in inducing MSPC needs further research to elucidate.

Currently, most research suggests that appropriate exercise contributes to recovery from musculoskeletal pain. Engaging in excessive physical activities that do not match one’s fitness level can increase the risk of chronic pain (21). Our study indicates that individuals with longer exercise times have a lower probability of developing MSPC (*p* < 0.001), highlighting the importance of physical exercise for preventing MSPC (21). Although it is widely known that exercise helps alleviate musculoskeletal pain (22), notably, the intensity and type of exercise for each individual still need to be determined, as blindly pursuing exercise duration may not be wise.

Recent research has highlighted a concerning trend: males experiencing post COVID symptoms seem to be particularly vulnerable to enduring health issues and experiencing worse health outcomes. This insight underscores the importance for public health policymakers to prioritize the health needs of this demographic (23). While our study did not identify gender and age as determinant factors for the MSPC, it does reveal a higher susceptibility for males to *de novo* musculoskeletal pain or to see an exacerbation of existing conditions (Supplementary Table S2), challenging the conventional belief that females are more prone to such pain (24, 25). That might be attributed to males consuming more alcohol during the COVID-19 period, as individuals who consume more alcohol have a 1.518 times higher risk of *ex novo* musculoskeletal pain compared to non-drinkers. The results also indicate that individuals who consume more alcohol have an increased likelihood of *ex novo* musculoskeletal pain (*p* = 0.001), similar to a previous study (26). Other literature suggests that over half of alcohol-dependent individuals experience some form of persistent pain, and alcohol may induce neuropathic changes in mice (27). Therefore, further research is indeed needed to explore the specific relationship between gender and post-COVID-19 musculoskeletal pain.

Furthermore, our results show that electronic device duration also plays an important role in the process of *ex novo* musculoskeletal pain (*p* = 0.035). Prolonged use of electronic devices often leads to excessive forward leaning of certain skeletal structures, placing additional stress and tension on surrounding muscles, resulting in prolonged muscle tension (28). Thus, maintaining healthy lifestyle habits is necessary.

As we consider the role of age, our findings present a counterintuitive trend (Supplementary Tables S2, S3). As age increases, our results show a decrease in the likelihood of suffering from *ex novo* musculoskeletal pain (*p* < 0.001) and exacerbation of musculoskeletal pain after COVID-19 infection (*p* < 0.001), which seems contradictory to previous research, a nationwide exploratory cross-sectional study identified older age as a significant risk factor for the onset of new widespread pain following a COVID-19 infection (29, 30). This might be because younger individuals tend to spend more time using electronic devices and this discrepancy may reveal complex biological and sociological factors that require further exploration. Furthermore, our research points to a correlation between the duration of walking and the risk of developing MSPC (*p* < 0.001). It appears that extended walking can induce fatigue in the soft tissues surrounding the knee joint, including muscles and ligaments. This finding emphasizes the necessity for a balanced approach to physical activity, suggesting that excessive walking could potentially precipitate MSPC and should be moderated accordingly.

Since the emergence of COVID-19, the development of vaccines has been a cornerstone of pandemic response efforts. The rapid global vaccination campaign has led to a significant reduction in severe COVID-19 cases, hospitalizations, and deaths. However, people who have been vaccinated may still become infected and develop COVID-19; this phenomenon is known as a “breakthrough infection,” but it does not mean that the vaccine is ineffective. Current studies suggest that COVID-19 vaccines might have protective and therapeutic effects on long-term COVID-19 (31). Indeed, SARS-CoV-2 vaccination reduced infections and symptomatic cases, demonstrating its paramount value as a preventive tool for occupational and public health. The study by Liviero et al. (32) showed that unvaccinated healthcare workers (HCWs) had a higher risk of infection. Nevertheless, our study suggests that a higher number of vaccine doses is significantly associated with an increased likelihood of *ex novo* musculoskeletal pain (Supplementary Table S2). These findings were similar to the study by Mason et al. (33) in which they found myalgia/arthralgia to be one of the observed side effects, though vaccines were mostly safe with severe adverse effects rarely reported. The detailed mechanisms are yet to be elucidated. A possible explanation is the mutation of the virus, such as the emergence of the Omicron variants. Omicron has posed a significant challenge in controlling the spread of the virus, limited the efficacy of vaccines, and caused side effects. Another study has also indicated that vaccination status does not increase protection against the syndrome compared to natural infection or vaccination alone (34). Therefore, the role of vaccination in MSPC warrants further studies to identify.

In recent years, research has shown that individuals experiencing chronic pain in multiple body parts have a higher risk of developing dementia and a broader and faster decline in cognitive abilities, including memory, executive function, learning, and attention (35). Therefore, timely medical care is undoubtedly important for patients with MSPC. Moreover, during the COVID-19 pandemic, there was a substantial decline in the number of consultations and diagnoses in primary care, which was strongly related to female gender, perceived poor health status, and elevated levels of depression and anxiety (36). Univariable logistic regression analysis and multivariable logistic regression analysis indicate that income level, frequency and severity of MSPC episodes, and PSS-10 scores are factors associated with healthcare-seeking behavior in individuals with MSPC.

To the best of our knowledge, this is the first study to investigate the factors associated with healthcare-seeking behavior for MSPC. The results indicate that individuals with higher frequency and severity of MSPC have a more apparent willingness to seek medical care, and those with higher income levels have a better willingness to seek medical care. As a rapidly developing middle-income country, the primary healthcare system is not yet perfect, and the allocation of medical resources is uneven. Previous research has shown a relationship between the deterioration of chronic diseases and declining economic conditions during the COVID-19 pandemic, with a more pronounced impact on chronic disease management behaviors in low-income areas such as rural regions (37). Thus, economic status is an important factor for the prevention and treatment of chronic diseases. Our results show that the willingness to seek medical care is 3.929 times higher in high-income populations compared to low-income populations. In the future, the government should implement further medical policies to improve healthcare-seeking behavior among low-income populations (38). Additionally, individuals with higher PSS-10 scores have a 1.122 times higher willingness to seek medical care than those with lower scores, indicating that stress may increase patients’ willingness to seek medical care. Literature suggests that high PSS-10 scores may lead to illness anxiety, which encourages patients to seek medical care (39).

Many studies indicate that a significant proportion of individuals who have recovered from COVID-19 go on to experience long-term or chronic symptoms. A large-scale cross-sectional investigation among French adults suggests that persistent physical symptoms after COVID-19 infection should not be automatically ascribed to SARS-CoV-2; a complete medical evaluation may be needed to prevent erroneously attributing symptoms to the virus (40). Geographical factors such as altitude significantly affect the symptom profile after COVID-19 (41). This finding, along with early research suggesting that TRP channels may be involved in various complications associated with COVID-19, indicates that a variety of biological mechanisms and environmental factors may be at play (42). To develop effective strategies for managing these post-viral symptoms, further research should be conducted to better understand the underlying mechanisms at play.

Musculoskeletal pain significantly reduces work efficiency, lowers the quality of life, leads to chronic occupational disabilities, and poses significant health challenges for healthcare providers (5). After the COVID-19 pandemic, musculoskeletal chronic pain is increasing. The purpose of our study is to describe the prevalence of MSPC and identify the associating factors of MSPC in a random population of COVID-19 survivors. Besides, our study represents a pioneering effort to examine the factors that are associated with healthcare-seeking behavior among individuals experiencing MSPC. These findings are expected to assist researchers and healthcare policymakers in developing effective prevention and control policies to prevent the prevalence of MSPC.

However, this study has several limitations that need to be acknowledged. Firstly, it was conducted only among a subset of the population in Fujian Province, and the results may not be entirely generalizable to other regions. Secondly, the use of an online electronic survey, while allowing for a broad reach, may have resulted in a selection bias toward individuals who are more technologically adept and have regular access to the internet. This could have excluded certain demographic groups, particularly the older adults or those in lower socioeconomic strata, who might have different experiences and outcomes related to COVID-19. Additionally, our study utilized a cross-sectional design, which allows for establishing associations between exposures and outcomes but cannot infer causality. Besides, the cross-sectional study may introduce potential bias, as participants’ responses are subject to personal perceptions, recall accuracy, and subjective interpretations of their symptoms and experiences. Lastly, we did not explicitly clarify the number and time of previous SARS-CoV-2 infections among participants prior to the study. Given the objective of our research to assess the prevalence of MSPC and its associating factors, understanding the history of infections could provide additional insights into the long-term effects of the virus on musculoskeletal pain. Future research should include more rigorously designed prospective studies to further explore these relationships. Despite these limitations, our study offers valuable insights into the prevalence and potential determinants of MSPC and contributes to the comprehension of it.

## Conclusion

In conclusion, a significant proportion of individuals experience MPSC. Several factors were found to be associated with MSPC, including education level, concomitant symptoms and PSS-10 score. The intention to seek medical treatment is very low and was associated with income level, severity, and frequency of MSPC.

## Data availability statement

The original contributions presented in the study are included in the article/Supplementary material, further inquiries can be directed to the corresponding author.

## Ethics statement

The studies involving humans were approved by The Institutional Ethics Committee of First Affiliated Hospital of Fujian Medical University. The studies were conducted in accordance with the local legislation and institutional requirements. Written informed consent for participation was not required from the participants or the participants’ legal guardians/next of kin because this is an online survey, and the informed consent was obtained from all the study subjects before enrollment.

## Author contributions

HL: Investigation, Writing – original draft. SZ: Investigation, Writing – review & editing. YL: Writing – review & editing, Software. MH: Writing – review & editing, Methodology. WZ: Writing – review & editing, Data curation. XZ: Writing – review & editing, Methodology, Supervision. YZL: Writing – review & editing, Supervision. CZ: Methodology, Supervision, Funding acquisition, Writing – review & editing.
