# Supplementary material for: The impact of COVID-19 infection on musculoskeletal pain and its associating factors: a cross-sectional study
Source: Front Public Health. 2024 Aug 27;12:1422659. doi: 10.3389/fpubh.2024.1422659 (PMC11384986; doi:10.3389/fpubh.2024.1422659)
Supplement: Supplementary file 2 [file Table_1.docx]

**Table S1.** The correlation between different associated factors

| Variables | Variables | Correlation Coefficient | P-value |
| --- | --- | --- | --- |
| Gender | Drinking | 0.252 | < 0.001 |
| Age | Electronic devices usage | - 0.178 | < 0.001 |
| PSS-10 | Healthcare-seeking intention | 0.192 | < 0.001 |

PSS-10: The Perceived Stress Scale

**Table S2.** The factors associated with the ex novo musculoskeletal pain after COVID-19

| Variables | Univariable analysis (P-value) | Multivariable  analysis (P-value) | OR [95% CI] |
| --- | --- | --- | --- |
| Gender | < 0.001 | < 0.001 | 2.304 [1.628 - 3.261] |
| Age | < 0.001 | < 0.001 | 0.730 [0.617 - 0.865] |
| BMI | 0.477 |  |  |
| Ethnicity | 0.041 | 0.346 | 1.904 [0.499 - 7.269] |
| Smoking | < 0.001 | 0.083 | 1.261 [0.970 - 1.640] |
| Drinking | < 0.001 | 0.006 | 1.518 [1.125 - 2.049] |
| Educational level | < 0.001 | < 0.001 | 2.885 [1.944 - 4.282] |
| Income level | 0.849 |  |  |
| Lifestyle |  |  |  |
| Exercise | 0.750 |  |  |
| Sleep | 0.001 | 0.602 | 1.072 [0.826 - 1.390] |
| Standing | 0.092 | 0.752 | 1.041 [0.809 - 1.340] |
| Sit (study or work） | 0.003 | 0.972 | 0.996 [0.814 - 1.219] |
| Walk | < 0.001 | 0.009 | 1.513 [1.110 - 2.062] |
| Electronic devices usage | 0.030 | 0.035 | 1.222 [1.014 - 1.472] |
| PSS-10 | 0.169 |  |  |
| Vaccination status | < 0.001 | 0.012 | 1.579 [1.105 - 2.256] |
| Number of concomitant symptoms | < 0.001 | < 0.001 | 1.284 [1.151 - 1.433] |

OR: odds ratios; 95%CI: 95% confidence intervals; BMI: Body Mass Index; PSS-10: The Perceived Stress Scale

**Table S3.** The factors associated with the exacerbation of musculoskeletal pain after COVID-19

| Variables | Univariable analysis (P-value) | Multivariable  analysis (P-value) | OR [95% CI] |
| --- | --- | --- | --- |
| Gender | 0.034 | < 0.001 | 2.972 [1.959 - 4.510] |
| Age | < 0.001 | 0.014 | 0.971 [0.949 - 0.994] |
| BMI | 0.111 |  |  |
| Ethnicity | 0.032 | 0.741 | 0.805 [0.223 - 2.905] |
| Smoking | 0.043 | 0.212 | 0.812 [0.586 - 1.126] |
| Drinking | 0.116 |  |  |
| Educational level | 0.091 | 0.052 | 1.811 [0.994 - 3.297] |
| Income level | < 0.001 | 0.131 | 0.776 [0.559 - 1.078] |
| Lifestyle |  |  |  |
| Exercise | < 0.001 | < 0.001 | 0.397 [0.280 - 0.563] |
| Sleep | 0.001 | 0.060 | 0.781 [0.603 - 1.011] |
| Standing | 0.298 |  |  |
| Sit (study or work） | 0.001 | 0.576 | 0.951 [0.799 - 1.133] |
| Walk | 0.001 | 0.893 | 0.977 [0.702 - 1.361] |
| Electronic devices usage | 0.001 | 0.095 | 0.855 [0.711 - 1.028] |
| PSS-10 | < 0.001 | 0.040 | 0.952 [0.907 - 0.998] |
| Vaccination status | 0.105 |  |  |
| Number of concomitant symptoms | < 0.001 | < 0.001 | 1.547 [1.353- 1.769] |

OR: odds ratios; 95%CI: 95% confidence intervals; BMI: Body Mass Index; PSS-10: The Perceived Stress Scale
